# Rho Kinase Enhances Contractions of Rat Mesenteric Collecting Lymphatics

**DOI:** 10.1371/journal.pone.0094082

**Published:** 2014-04-07

**Authors:** Kristine H. Kurtz, Flavia M. Souza-Smith, Andrea N. Moor, Jerome W. Breslin

**Affiliations:** 1 Department of Physiology, School of Medicine, Louisiana State University Health Sciences Center, New Orleans, Louisiana, United States of America; 2 Department of Molecular Pharmacology and Physiology, Morsani College of Medicine, University of South Florida, Tampa, Florida, United States of America; University of Missouri, United States of America

## Abstract

The mechanisms that control phasic and tonic contractions of lymphatic vessels are poorly understood. We hypothesized that rho kinase ROCK, previously shown to increase calcium (Ca^2+^) sensitivity in vascular smooth muscle, enhances lymphatic contractile activity in a similar fashion. Contractions of isolated rat mesenteric lymphatic vessels were observed at a luminal pressure of 2 cm H_2_O in a 37°C bath. The expression of ROCK in isolated rat mesenteric lymphatic vessels was assessed by Western blotting and confocal microscopy. The role of ROCK in contractile function was tested using two specific yet structurally distinct inhibitors: H1152 (0.1–10 μM) and Y-27632 (0.5–50 μM). In addition, lymphatics were transfected with constitutively active (ca)-ROCK protein (2 μg/ml) to assess gain of contractile function. Vessel diameter and the concentration of intracellular free Ca^2+^ ([Ca^2+^]_i_) were simultaneously measured in a subset of isolated lymphatics loaded with the Ca^2+^-sensing dye fura-2. The results show expression of both the ROCK1 and ROCK2 isoforms in lymphatic vessels. Inhibition of ROCK increased lymphatic end diastolic diameter and end systolic diameter in a concentration-dependent manner. Significant reductions in lymphatic tone and contraction amplitude were observed after treatment 1–10 μM H1152 or 25–50 μM Y-27632. H1152 (10 μM) also significantly reduced contraction frequency. Transient increases in [Ca^2+^]_i_ preceded each phasic contraction, however this pattern was disrupted by either 10 μM H1152 or 50 μM Y-27632 in the majority of lymphatics studied. The significant decrease in tone caused by H1152 or Y-27632 was not associated with a significant change in the basal [Ca^2+^]_i_ between transients. Transfection with ca-ROCK protein enhanced lymphatic tone, but was not associated with a significant change in basal [Ca^2+^]_i_. Our data suggest that ROCK mediates normal tonic constriction and influences phasic contractions in lymphatics. We propose that ROCK modulates Ca^2+^ sensitivity of contractile proteins in lymphatics.

## Introduction

Lymphatics play a critical role in normal cardiovascular function, tissue fluid homeostasis, inflammation, adaptive immunity, digestive lipid uptake, metabolism, and the regulation of salt storage [1], [2]. Individuals with dysfunctional lymphatic vessels often suffer from chronic edema and impaired immune responses [3]. The most recognizable form of lymphatic dysfunction is lymphedema, which can vary from mild swelling to a severe, disfiguring and debilitating disease.

The intrinsic pumping action of collecting lymphatics drives normal lymph flow, and is generated by their smooth muscle layer. Lymphatic pumping consists of a phasic, cardiac-like contractile cycle, superimposed over smooth muscle-like tone between the phasic contractions [4]. As in other muscle types, the rise and fall in cytosolic free Ca^2+^ ([Ca^2+^]_i_) is considered the principal mechanism that initiates contraction and relaxation, respectively [5]. In collecting lymphatics, each phasic contraction is immediately preceded by a transient rise in [Ca^2+^]_i_, while a certain basal [Ca^2+^]_i_ between contractions helps maintain tone [6]–[8].

For the maintenance of tone in smooth muscle, Ca^2+^ binds to calmodulin, and this complex activates the catalytic subunit of myosin light-chain kinase (MLCK). In turn, MLCK phosphorylates Ser19 and Thr18 on the regulatory myosin light chain (MLC) [5], activating the myosin ATPase, leading to contraction. A fall in [Ca^2+^]_i_ inactivates MLCK and permits dephosphorylation of MLC by myosin light chain phosphatase (MLCP). A role for MLCK in establishing tone and phasic contractions in collecting lymphatics and the thoracic duct has previously been demonstrated [9],[10].

In addition, the contractile mechanisms in smooth muscle display a varying Ca^2+^ sensitivity in response to a variety of agonists, defined as the ability to change the level of tone generated at a given level of [Ca^2+^]_i_ [11]. Increases in Ca^2+^ sensitization in response to various agonists are thought to involve G-protein coupled inhibition of MLCP, shifting the kinase/phosphatase balance in favor of MLCK so that a higher level of MLC phosphorylation is achieved at a given [Ca^2+^]_i_ [11]–[13]. The inhibition of MLCP could be mediated by either direct binding and inhibition of protein kinase C (PKC)-potentiated phosphatase inhibitor of 17 kDa (CPI-17), or phosphorylation of the MLCP by Rho kinase (ROCK) [12]–[15]. Notably, application of the ROCK inhibitor Y-27632 has been shown to cause a loss of tone in isolated rat iliac collecting lymphatic vessels and in the rat thoracic duct [16],[17]. In addition, mesenteric collecting lymphatics isolated from a rat acute alcohol intoxication model display a relaxed phenotype that has been associated with decreased levels of the active, GTP-bound form of RhoA [18]. This phenotype was rescued by experimentally enhancing ROCK activity with a protein transfection method [18].

What remains unclear is whether the reduction in tone in response to ROCK inhibitors represented a change in Ca^2+^ sensitivity. In addition, the expression of ROCK in lymphatic tissues has not previously been established. The goal of these studies was to test the hypothesis that ROCK increases Ca^2+^ sensitivity, leading to enhanced tone in isolated collecting lymphatic vessels. We performed studies to determine whether both ROCK isoforms are present in lymphatic smooth muscle cells. We also evaluated how pharmacologic inhibition of ROCK affects lymphatic [Ca^2+^]_i_ and the lymphatic contractile cycle. Lastly, we investigated how experimentally enhancing ROCK activity affects [Ca^2+^]_i_ and lymphatic pumping.

## Methods

### Ethics Statement

All animal procedures were carried out in strict accordance as outlined in the National Institute of Health Guide for the Care and Use of Laboratory Animals (8^th^ Edition, 2011). The protocol was approved by the Louisiana State University Health Sciences Center – New Orleans Institutional Animal Care and Use Committee (permit number: 2875) and the University of South Florida Institutional Animal Care and Use Committee (permit number: RIS00000097). Rats were anesthetized with a mixture of ketamine (90 mg/kg) and xylazine (9 mg/kg) i.m. prior to surgery.

### Animals

Male Sprague-Dawley rats (275–350 g body weight) were housed in a controlled temperature (22°C) and controlled illumination (12∶12 h light dark cycle) environment. After arrival, the rats were submitted to a one-week acclimation period and were provided standard rat chow (2018 Teklad Global 18% Protein Rodent Diet, Harlan) and water *ad libitum*.

### Collecting Lymphatic Isolation

After anesthesia was induced in the rat, a midline laparotomy was performed, and the small intestine and mesentery were exteriorized and excised and placed in ice-cold albumin physiological salt solution (APSS: NaCl, 120 mM; KCl, 4.7 mM; CaCl_2_·2H_2_O, 2 mM; MgSO_4_·7H_2_O, 1.2 mM; NaH_2_PO_4_, 1.2 mM; Na pyruvate, 2 mM; glucose, 5 mM; EDTA, 0.02 mM; MOPS, 3 mM and purified BSA 1 g/100 ml). Rats were euthanized with Euthasol/Somnasol (0.1 ml/450 g body weight, i.m.) followed by opening of the chest to confirm death. In each experiment, a section of mesentery was pinned in a dissection chamber containing ice-cold APSS, and with the aid of a stereomicroscope, a collecting lymphatic vessel (60–200 μm internal diameter and 0.5–1.0 cm in length) was carefully dissected from surrounding adipose and connective tissue. The isolated lymphatic was transferred to an isolated vessel chamber (Living Systems Instrumentation, Burlington, VT) and was mounted onto two resistance-matched glass micropipettes containing APSS and secured with nylon thread. The vessels typically contained at least two valves, so that one complete lymphangion was available for study. The chamber was transferred to a Nikon Eclipse TS100 inverted microscope, equipped with a halogen lamp, 10× objective, CCD camera, DVD recorder, and a Living Systems Video Dimension Analyzer for video image acquisition, storage, and lymphatic diameter measurement. Adjustable reservoirs containing APSS, on a wall-mounted manometer, were connected to the pipettes to set the intraluminal pressure. All experiments were performed with the bath temperature set at 37°C and the reservoirs set at a height of +2 cm H_2_O relative to the isolated lymphatic. This luminal pressure is within the normal physiological range for lymphatics [19]. The vessels were allowed to equilibrate for 30–45 minutes in order to establish baseline contractions. Only lymphatic vessels that displayed phasic contractions that reduced internal diameter during systole by at least 25% of the diastolic diameter were used for these studies.

### Experimental Protocols

ROCK activity was blocked using H1152 added to the bath at final concentrations of 0.1, 1 and 10 μM, or Y-27632 at 0.5, 5.0, 25, and 50 μM (EMD-Millipore, Billerica, MA). These concentrations ranges were chosen based on the reported IC50 values of 2.5 μM for H1152 and 12.8 μM for Y-27632 to inhibit ROCK-mediated phosphorylation in cultured cells [20]. Both inhibitors were dissolved in APSS. For both inhibitors, the concentration was raised by successive additions to the bath every 20 minutes. Vessel diameter was tracked throughout each experiment.

The following parameters were determined from the lymphatic luminal diameter measurements [6],[21]: Contraction frequency (CF), end diastolic diameter (EDD), end systolic diameter (ESD), amplitude of contraction (AMP  =  EDD-ESD), the maximal passive diameter (MaxD; determined in Ca^2+^-free APSS), tone  =  100 * (MaxD – EDD)/MaxD, and Ejection Fraction (EF)  =  (EDD^2^-ESD^2^)/(EDD^2^). EDD, ESD, and AMP were normalized to MaxD to account for variability in the resting diameter of lymphatics. For baseline data, the means presented for each parameter represent the averages for the 5-min. period just prior to the addition of inhibitors. For each concentration of the ROCK inhibitors, the 5-min. increment after the first 2 min. post addition was used to calculate the mean data (i.e., means represent 2–7 min. after addition of each concentration).

### Measurement of [Ca^2+^]_i_ in Isolated Lymphatics

The protocol for measuring [Ca^2+^]_i_ in isolated lymphatics has been previously described in detail [6],[18]. Briefly, isolated lymphatics were loaded with the ratiometric, Ca^2+^-sensing dye fura-2-acetoxymethyl ester (AM) (Molecular Probes, Eugene, OR) by exchanging the bath solution to APSS containing fura-2 AM (2 μM) and pluronic acid (0.2% wt/vol) for 30 minutes at 37°C. The fura-2 AM was only added to the abluminal side to restrict loading to the smooth muscle layer. The bath solution was then changed twice to normal APSS to wash out extracellular fura-2. The vessel was allowed to equilibrate for at least 20 min. to allow re-establishment of spontaneous contractions. A Lambda LS 300W Xenon lamp equipped with a LS-filter wheel (Sutter Instrument Co., Novato, CA) was used to alternately illuminate the vessel at 340 and 380 nm wavelengths for durations of 50 ms each. The emission light was passed through a band-pass filter (510-nm; Chroma Technology Corp., Bellows Falls, VT) with a bandwidth of 80 nm and detected by a Photometrics HQ^2^ camera (Roper Scientific, Sarasota, FL). The acquisition software used to capture images and for data analysis was Nikon Elements 4.0 (Nikon Instruments, Melville, NY).

Changes in both vessel diameter and the ratio of fluorescence at 340 nm and 380 nm (340/380), indicative of [Ca^2+^]_i_, were measured. To determine the 340/380 ratio, a rectangular region of interest that included the entire lymphatic vessel and surrounding area was selected, so that the vessel was fully tracked during relaxation and contraction. We were careful to exclude regions on lymphatic vessels where there appeared to be a capillary attached to the exterior, as our target was to estimate smooth muscle [Ca^2+^]_i_. An increase in 340/380 indicates an increase in [Ca^2+^]_i_. Using the 380 nm channel of the same image set, we used a contrast threshold protocol in Nikon Elements to track the vessel within a region of interest around a short segment of the vessel. This allowed us to automatically measure the distance between outer edges of the vessel. The resulting external diameter data was then exported to Microsoft Excel for further analysis. All [Ca^2+^]_i_ data are expressed using the raw 340/380 ratio values [22].

### Protein Transfection of Lymphatic Vessels

ROCK activity was experimentally enhanced in isolated lymphatics by transfecting constitutively active (ca)-ROCK protein, a purified, recombinant truncated form of ROCK2 lacking the regulatory domain (Millipore), with a recently developed lymphatic protein transfection protocol [18]. Using recombinant EGFP (Cell Biolabs, San Diego, CA), we investigated whether applying the polyamine transfection reagent TransIT-LT1 (Mirus, Madison, WI) plus EGFP shuttles the protein mainly into the smooth muscle layer of isolated lymphatics. Rat mesenteric lymphatic vessels were isolated, cannulated and pressurized at 2 cm H_2_O, warmed to 37°C, and phasic contractions were allowed to develop. At the same time, TransIT-LT1 (40 μL) was mixed with EGFP protein (10 μg in a 10 μL volume) and incubated at room temperature for 30 min. The 50 μL transfection mix was then added to 5 mL of APSS warmed to 37°C. The suffusion bath contents were replaced with the APSS containing final concentrations of 8 μL/mL TransIT-LT1 and 2 μL/mL EGFP. After a 30-min. incubation at 37°C, the isolated lymphatic was fixed in 4% paraformaldehyde for 15 min. at room temperature, followed by 3 washes with Ca^2+^/Mg^2+^-free Dulbecco's PBS (CMF-DPBS). The lymphatic vessel was then mounted on glass slides with Secure-Seal imaging spacers and Prolong Gold anti-fade reagent with DAPI (Molecular Probes, Eugene, Oregon), under a #1 glass coverslip for laser confocal imaging (see below).

The protocol to experimentally enhance ROCK in the lymphatic vessels was essentially the same, except that recombinant constitutively active (ca)-ROCK protein (10 μg in 10 μl volume) was added to 40 μL TransIT-LT1. Baseline lymphatic diameter data was evaluated just prior to, and 30 min. after the addition of the APSS containing TransIT-LT1 and ca-ROCK. Addition of TransIT-LT1 alone (8 μl/ml final) served as a control for the lymphatic diameter studies.

### Immunofluorescence Labeling and Confocal Imaging of Isolated Lymphatics

Freshly isolated rat mesenteric lymphatics were transferred to custom chambers for fixation and labeling. Initially each chamber was filled with APSS. One end of each lymphatic was mounted and tied to a glass micropipette cannula and the other end was left free to float in the bath. This allowed for bath solution to be drawn into the vessel lumen by gently applying negative pressure on the micropipette. In some cases a luminal valve restricted flow and in this case the vessel had to be removed and remounted onto the cannula between certain steps. Also, a small amount of luminal pressure was applied from time to time to prevent vessel collapse during the labeling protocol, with solutions appropriate to the various steps. The micropipette and bath initially contained APSS containing 1 μg/ml TRITC-conjugated BSI-Lectin (Sigma L5264) that was gently perfused through the mounted lymphatic, which was then incubated at room temperature for 30 min. The lymphatic was then rinsed with CMF-DPBS four times for five minutes. Next, the vessel was fixed with 4% paraformaldehyde for 10–15 min. at room temperature, followed by two 5-min. washes with 100 mM glycine buffer and one 5-min. wash with CMF-DPBS. Ice-cold acetone was applied for 5 min. to permeabilize the cell membranes, followed by three 5-min. washes with CMF-DPBS. A blocking solution consisting of 5% normal goat serum in CMF-DPBS was applied for 30 min. at room temperature, followed by overnight incubation with primary antibodies in antibody dilution buffer (151 mM NaCl, 17 mM trisodium citrate, 2% goat serum, 1% BSA, 0.05% Triton X-100, 0.02% NaN_3_) at 4°C: 1∶6000 mouse anti-α/**γ**-smooth muscle actin (Millipore Mab1522); and either 1∶50 rabbit anti-ROCK1 (Santa Cruz sc-5560) or 1∶1000 rabbit anti-ROCK2 (Abcam ab66320). Labeling controls received antibody dilution buffer containing no primary antibody. After overnight incubation, three 10-min. rinses with antibody wash solution (151 mM NaCl, 17 mM trisodium citrate, 0.05% Triton X-100) were performed. The vessels were then incubated for 30 minutes at room temperature with antibody dilution buffer containing secondary antibodies: 1∶100 Alexa-488-goat anti-rabbit IgG (Molecular Probes A11008) and 1∶100 Alexa-635-goat anti-mouse IgG (Molecular Probes A31574). Three 10-min. rinses with antibody wash solution were performed, and each vessel was removed from its cannula, placed on a glass slide with a Secure-Seal imaging spacer in 10 μl of Prolong Gold Anti-Fade reagent containing DAPI, and covered with a #1 glass coverslip. Care was taken to keep the lymphatic vessel lumen patent. Confocal z-stack images of the vessels were obtained with an Olympus FV1000 MPE multiphoton laser-scanning microscope, using a 40× objective (UPLFLN, NA 0.75), at the Lisa Muma Weitz Advanced Microscopy and Cell Imaging Core at the University of South Florida. Movies (AVI format) of the image stacks of the lymphatic vessels were generated with FIJI/ImageJ open source imaging software (http://fiji.sc).

### Western Blotting

Approximately 10 lymphangions were isolated from rat mesentery, placed in 200 μl of ice-cold 1× RIPA containing HALT Protease and Phosphatase Inhibitor Cocktail (Pierce, Rockford, IL), and the mixture was sonicated at 4°C twice for 5 s with a Fisher Sonic Dismembranator, model FB-120 (Fisher Scientific, Asheville, NC). Protein concentrations were determined with the BCA protein assay (Pierce), and lysate was mixed with 4× NuPage LDS sample buffer containing reducing agent (Invitrogen – Life Technologies, Grand Island, NY). Proteins were separated by SDS-PAGE in 4–20% Novex Bis-Tris gels (Invitrogen), with 20 μg of protein loaded per lane. The NexusPointer prestained protein ladder (BioNexus, Oakland, CA) was used to determine molecular weight vs. mobility. Proteins were transferred to Immobilon-P PVDF membranes (Millipore) by wet transfer. Membranes were blocked with 5% BSA in TBST. Primary antibodies were 1∶100 rabbit anti-ROCK1 (Santa Cruz sc-5560) and 1∶500 rabbit anti-ROCK2 (Abcam ab66320). The secondary antibody was 1∶3000 donkey anti-rabbit IgG-HRP (Abcam ab97064). Bands were visualized using WestPico Supersignal reagent (Pierce; 5 min. incubation) and a BioRad Chemi Doc XRS+ System with Quantity 1-D Analysis Software (5 min. exposure).

### Data Analysis

Representative tracings are shown for the ROCK inhibitor protocols. Summarized data are presented as mean ± SE. The responses of lymphatic vessels over time to various treatments were evaluated with repeated measures ANOVA, followed by Dunnett's test to compare individual time points to baseline when appropriate. In addition, concentration-response relationships were investigated using nonlinear regression analysis. Data were normalized to the absolute percentage values based on the maximum (Max) and minimum (Min) responses observed, and curves were fit to the equation Y  =  Max + [(Max – Min)/(1 + 10^logEC50-X^)] using GraphPad Prism 6 software. When comparing two time-matched groups, two-way repeated-measures ANOVA followed by Bonferroni t-tests to compare groups at individual time points was the analysis of choice. For a single comparison between baseline and treatment, a paired t-test was used. Significance was accepted at P<0.05.

## Results

### Expression and localization of ROCK1 and ROCK2 in mesenteric lymphatic vessels

**Figure 1:**
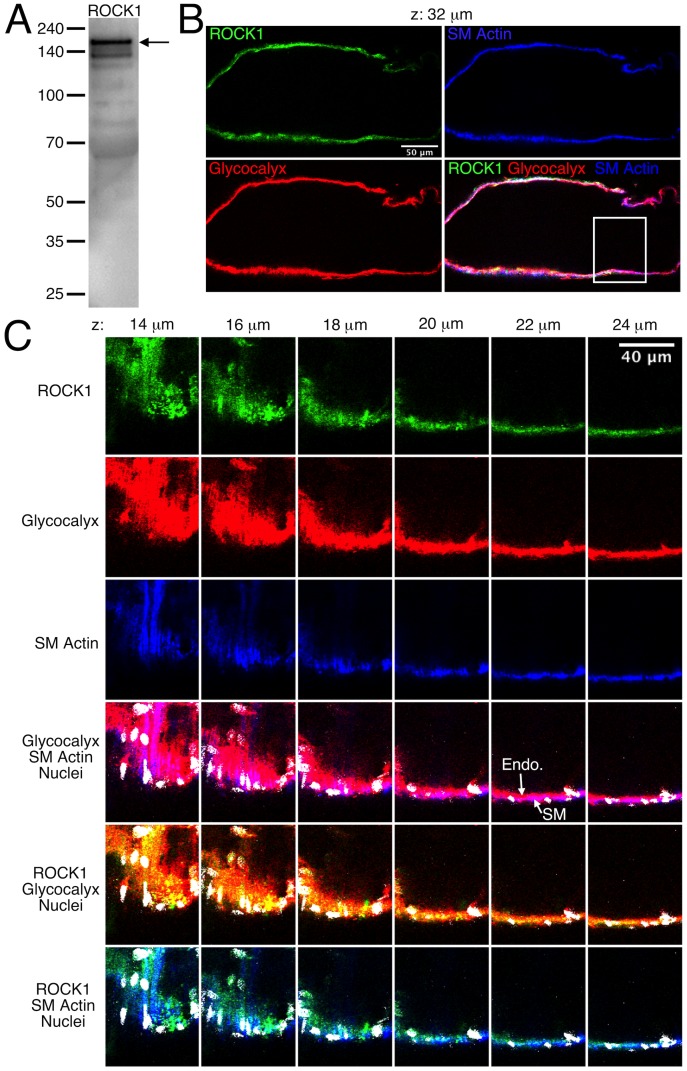
Expression of ROCK1 in rat mesenteric lymphatic vessels. *A.* Western blot for ROCK1. *B.* Confocal section images of the isolated lymphatic vessel shown in Movie S1. The vessel was labeled to identify ROCK1 (green), glycocalyx that is present on both the endothelial and smooth muscle layers (red), and smooth muscle (SM) actin to distinguish the smooth muscle layer (blue). An overlay of the green, red, and blue channels is also shown. The confocal series included slices taken every 2 μm, and the panel *B* images correspond to the confocal slice taken 32 μm from the start of the series. The white box in the overlay image indicates the zoom-in area shown in panel *C*, where images corresponding to confocal slices at 14–24 μm through the series are shown. The ROCK1 (green), glycocalyx (red), and smooth muscle actin (blue) channels, along with overlays including nuclei (white) are shown. For the glycocalyx/smooth muscle actin overlay, magenta areas represent overlap of the red and blue labels. This helps distinguish the endothelium (Endo.), which labels only in red, from the smooth muscle (SM) layer, which is blue/magenta. For the ROCK1/glycocalyx overlay, yellow areas represent overlap. For the ROCK1/SM actin overlay, cyan areas represent overlap. Representative of three experiments.

**Figure 2:**
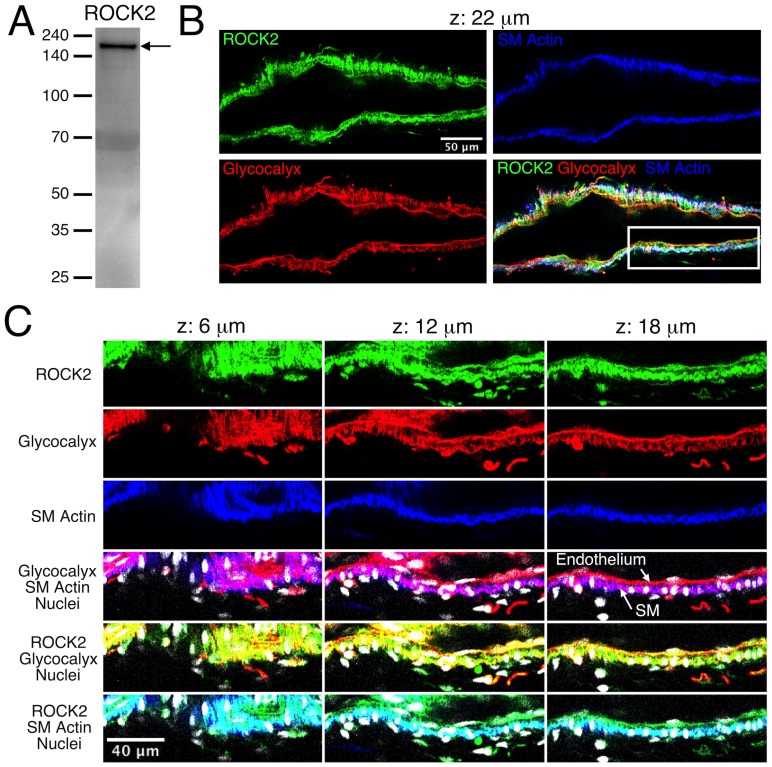
Expression of ROCK 2 in rat mesenteric lymphatic vessels. *A.* Western blot for ROCK2. *B.* Confocal section images of the isolated lymphatic vessel shown in Movie S2. The vessel was labeled to identify ROCK2 (green), glycocalyx that is present on both the endothelial and smooth muscle layers (red), and smooth muscle (SM) actin to distinguish the smooth muscle layer (blue). An overlay of the green, red, and blue channels is also shown. The confocal series included slices taken every 2 μm, and the panel *B* images correspond to the confocal slice taken 22 μm from the start of the series. The white box in the overlay image indicates the zoom-in area shown in panel *C*, where images corresponding to confocal slices at 6, 12, and 18 μm through the series are shown. The ROCK2 (green), glycocalyx (red), and smooth muscle actin (blue) channels, along with overlays including nuclei (white) are shown. For the glycocalyx/smooth muscle actin overlay, magenta areas represent overlap of the red and blue labels. This helps distinguish the endothelium, which labels only in red, from the smooth muscle (SM) layer, which is blue/magenta. For the ROCK1/glycocalyx overlay, yellow areas represent overlap. For the ROCK1/SM actin overlay, cyan areas represent overlap. Representative of three experiments.

The expression and localization of ROCK1 and ROCK2 in rat mesenteric lymphatic vessels was determined using Western blotting and immunofluorescence labeling, respectively (Figs. 1 and 2). A strong band at ∼160 kDa was evident in the Western blot for ROCK1 using protein lysates from isolated rat mesenteric lymphatic vessels (Fig. 1*A*), along with some weaker, presumably non-specific bands at lower molecular weights. Fig. 1*B* shows a single confocal section of a lymphatic labeled to identify glycocalyx (both smooth muscle and endothelial), smooth muscle actin, ROCK1, and nuclei. The entire z-stack for this lymphatic vessel is shown in Movie S1. A close-up view of sections from the z-stack shows that ROCK1 labeling was present in endothelium, smooth muscle, and in some unidentified cells present on the abluminal side of the smooth muscle layer (Fig. 1*C*). ROCK2 was also identified in lymphatic vessel lysates by Western blotting (Fig. 2*A*), and ROCK2 labeling was prominent in both the smooth muscle and endothelial layers (Fig. 2*B*–*C* and Movie S2). Controls for immunolabeling showed little or no signal (data not shown). These findings show that ROCK1 and ROCK2 are present in rat mesenteric lymphatics, with both present in the smooth muscle layer and endothelium.

### Inhibition of ROCK reduces the tone and CF of rat mesenteric lymphatic vessels

**Figure 3:**
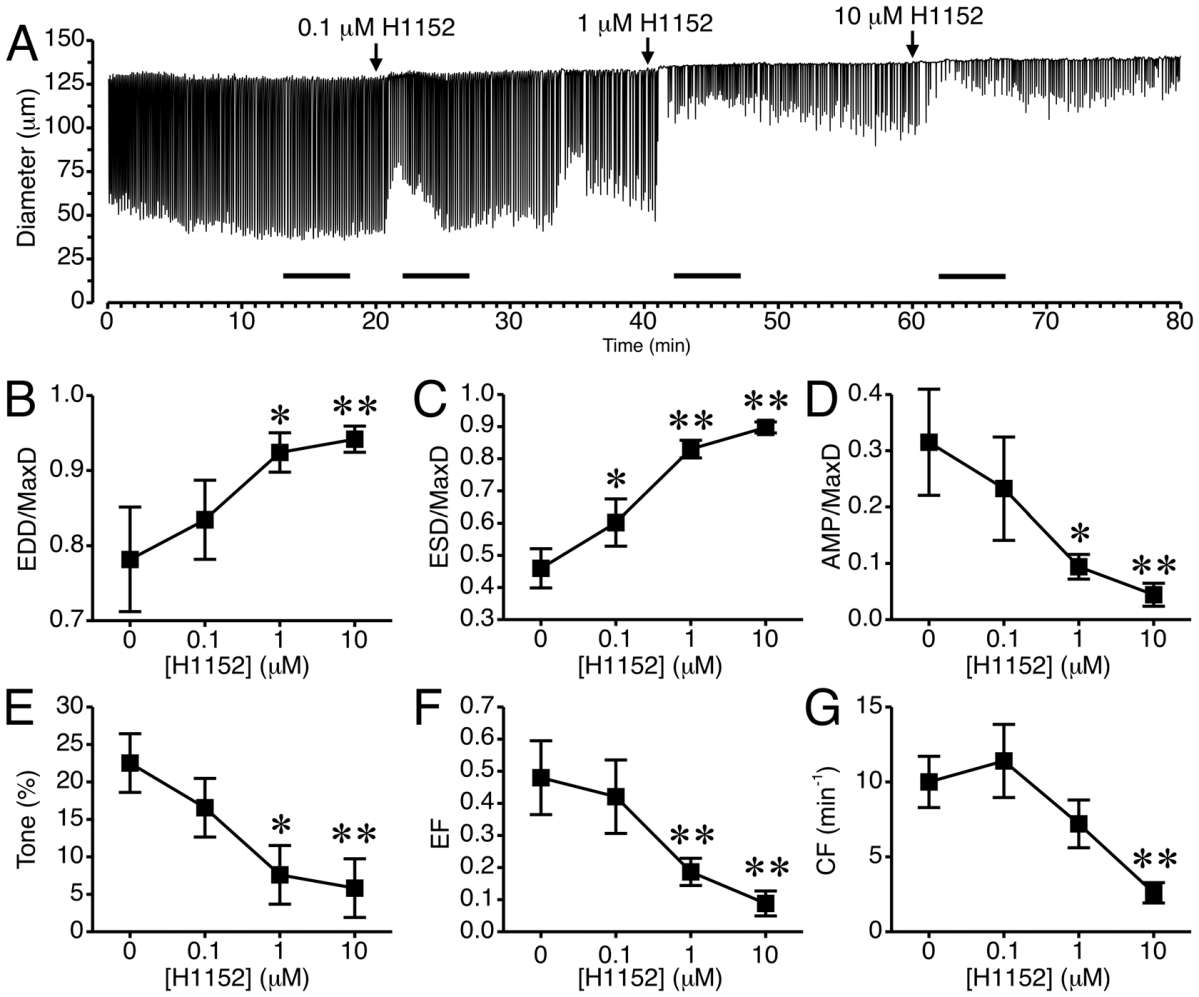
Inhibition of ROCK with H1152 causes relaxation of rat mesenteric collecting lymphatic vessels. *A.* An example of the lymphatic diameter over time before and after treatment with increasing concentrations of the ROCK Inhibitor H1152. Lines indicate the 5-min. intervals used for the data summarized in panels *B*–*G*. H1152 significantly increased EDD/MaxD (*B*) and ESD/MaxD (*C*) when applied at concentrations of 1 and 10 μM. *D*–*F.* AMP/MaxD, lymphatic tone, and EF were all significantly decreased by 1 and 10 μM H1152. *G.* H1152 applied at 10 μM significantly decreased CF. N = 5 lymphatics from 5 different rats. *P<0.05 and **P<0.01 vs. baseline prior to H1152 addition.

We tested the impact of the selective ROCK inhibitor, H1152, on lymphatic pumping (Fig. 3). The time course of changes in diameter for a representative lymphatic treated with H1152 at concentrations of 0.1, 1, and 10 μM is shown in Fig. 3*A*. For comparison, an untreated lymphatic is shown in Fig. S1. In the tracing shown in Fig. 3*A*, a noticeable increase in EDD occurred after each concentration of H1152 was added. There was also a noticeable transient increase in ESD after addition of 0.1 and 1 μM H1152. After 10 μM H1152 was added, further dilation and less frequent pumping were apparent. In some lymphatics phasic contractions were reduced to precontractions that were not strong enough to elicit changes in diameter and were thus not counted (data not shown). Summarized results from 5 different lymphatic vessels are shown in Fig. 3*B*–*G*. H1152 caused a concentration-dependent increase in mean normalized EDD and ESD (Fig. 3*B, C* and Fig S2). H1152 also caused concentration-dependent decreases in normalized AMP, tone, and EF (Fig. 3*D, E, F* and Fig S2). In addition, 10 μM H1152 significantly decreased CF (Fig. 3*G*).

**Figure 4:**
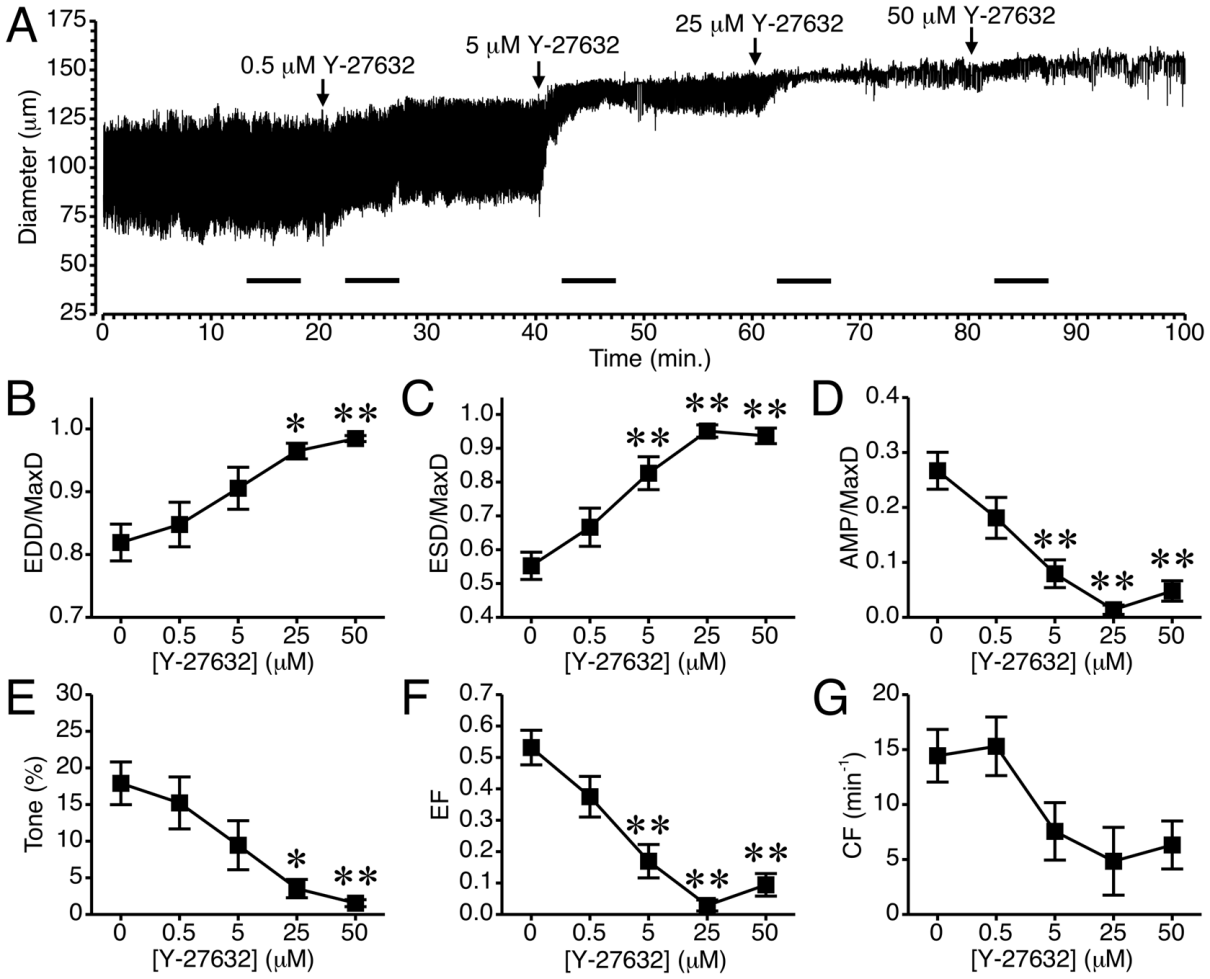
Inhibition of ROCK with Y-27632 causes relaxation of rat mesenteric collecting lymphatics. *A.* An example tracing of the lymphatic diameter over time before and after treatment with increasing concentrations of Y-27632. The bars in the time-course indicate the 5-minute intervals used for the data summarized in panels *B*–*G*. *B.* Application of 25–50 μM Y-27632 significantly increased mean EDD/MaxD. *C.* Y-27632 (5–50 μM) also significantly increased mean ESD/MaxD. *D.* AMP/MaxD significantly decreased with 5–50 μM Y-27632. *E.* Y-27632 (25–50 μM) significantly decreased lymphatic tone. *F.* Y27632 (5–50 μM) significantly decreased ejection fraction (EF). *G.* Y-27632 did not have a significant impact on CF. N = 10 lymphatics from 10 different rats were studied. The first 5 lymphatics were treated with only 0.5 and 5 μM Y-27632, and the second set of 5 lymphatics was treated with 0.5, 5, 25, and 50 μM Y-27632. *P<0.05 and **P<0.01 vs. baseline prior to Y-27632 addition.

The impact of a less potent, structurally distinct, yet more widely used ROCK inhibitor, Y-27632, on rat mesenteric lymphatic pumping was also tested (Fig. 4). The time course of changes in luminal diameter from a representative isolated lymphatic vessel exposed to 0.5–50 μM Y-27632 is shown in Fig. 4*A*. In this tracing, at each concentration of Y-27632 added there was a noticeable relaxation of the vessel, with slight increases in EDD, and starting with the 5 μM concentration, a marked decrease in the contraction amplitude. In some experiments, following application of Y-27632 at 5–50 μM, phasic contractions either ceased or were not strong enough to elicit a reduction in diameter. The summarized data from 10 lymphatics tested is shown in Fig. 4*B*–*G* and Fig. S3. With the addition of 25 and 50 μM Y-27632, there was a significant increase in mean normalized EDD compared to baseline (Fig. 4*B*). Addition of 5 μM Y27632 and higher concentrations significantly increased mean normalized ESD (Fig. 4*C*) and also significantly decreased mean normalized AMP (Fig. 4*D*). Y-27632 also significantly reduced lymphatic vessel tone at 25 and 50 μM (Fig. 4*E*), and ejection fraction at 5, 25, and 50 μM (Fig. 4*F*). In contrast, mean CF, although reduced to about half of the baseline value starting with the 5 μM concentration, was not significantly different from baseline at any of the time points tested (Fig. 4*G*). This was due in large part to variability in whether the isolated lymphatics continued to display phasic contractions after 5 μM or higher concentrations of Y-27632 were added.

### Ca^2+^ transients and lymphatic pumping during treatment with ROCK inhibitors

**Figure 5:**
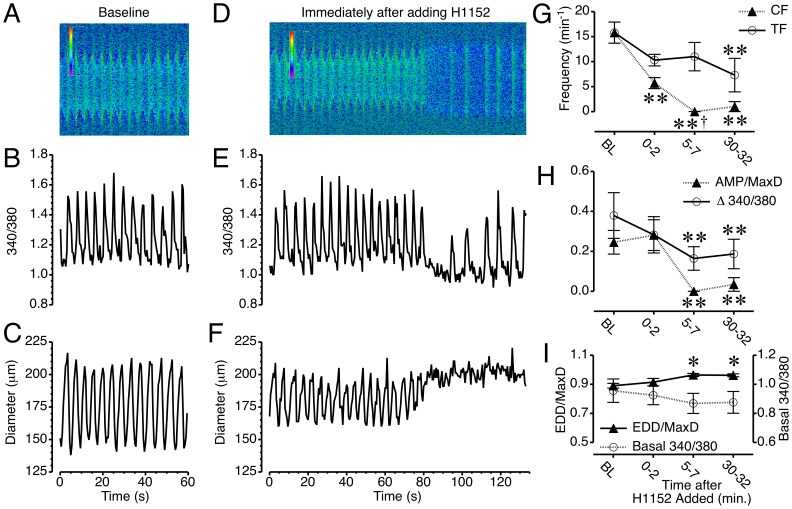
Ca^2+^ transients persist during H1152-induced inhibition of phasic contractions in mesenteric collecting lymphatics. *A.* Kymograph of the lymphatic shown in Movie S3, with the x-axis representing time, the y-axis representing distance, and the heat map representing the intensity of the 340/380 ratio. *B*–*C.* Changes in the 340/380 ratio and lymphatic diameter versus time. *D.* Kymograph of Movie S4, immediately after H1152 was added to the bath at a final concentration of 10 μM. *E*–*F* Changes in 340/380 ratio and lymphatic diameter versus time immediately after H1152 was added. *G*. Time course of the mean changes in CF and transient frequency (TF) in response to H1152. *H*. Time course of mean changes in AMP/MaxD and the Ca^2+^ transient magnitude (Δ340/380) after H1152 treatment. *I*. Time course of mean changes in EDD/MaxD and the basal (diastolic) 340/380 ratio between Ca^2+^ transients in response to H1152. *P<0.05 and **P<0.01 versus baseline. †P<0.05, CF vs. TF, same time point. N = 5 lymphatic vessels studied.

We next studied the relationship between [Ca^2+^]_i_ and changes in diameter, using isolated lymphatics loaded with the ratiometric Ca^2+^-sensing dye, fura-2. The overall contractile cycle was maintained in isolated mesenteric lymphatic vessels after loading with fura-2, with no significant changes in the lymphatic contractile parameters (Table 1). Simultaneous measurement of the 340/380 ratio and diameter showed a transient increase in [Ca^2+^]_i_ just prior to each phasic contraction (Movie S3, and Fig. 5*A*–*C*). The immediate impact of the ROCK inhibitor H1152 on Ca^2+^ transients and changes in diameter of the same isolated lymphatic vessel is shown in Movie S4, and Fig. 5*D*–*F*. About 1.5 min. after the addition of 10 μM H1152, there was a cessation of both Ca^2+^ transients and phasic contractions. Shortly after, the Ca^2+^ transients resumed, yet phasic contractions did not return (Fig. 5*D*–*F*). The summarized data for the five lymphatic vessels studied with this protocol (Fig. 5*G*) shows the disparity in Ca^2+^ transient frequency (TF) and CF that developed within the first two minutes after H1152 was added. For the two-minute period starting 5 min. after H1152 was added, all five vessels displayed no phasic contractions while Ca^2+^ transients persisted. At 30–32 min. after H1152 was added, phasic contractions returned in one of the five vessels studied, resulting in a slight increase in mean CF compared to the 5–7 min. period (Fig. 5*G*). These results indicate that the cessation of phasic contractions elicited by H1152 treatment was not due to a loss of Ca^2+^ transients.

**Table 1:** Impact of fura-2 loading on lymphatic pump parameters.

	Before Loading	After Fura-2 Loading
EDD/MaxD	0.89±0.05	0.89±0.02
ESD/MaxD	0.70±0.06	0.65±0.06
AMP/MaxD	0.19±0.04	0.25±0.06
CF	18.2±3.7	15.8±2.1

N = 5 lymphatics studied.

That being said, H1152 also affected the Ca^2+^ transients, which were notably less frequent, albeit insignificantly compared to baseline (Fig. 5*G*). In addition, the Ca^2+^ transients that persisted during the H1152-induced loss of phasic contractions had a significantly diminished magnitude at 5–7 and 30–32 min after H1152 was added to the bath (Fig. 5*H*). At the 5–7 min time point, the mean AMP was zero because none of the lymphatics displayed phasic contractions. The mean AMP at 30–32 min. also was significantly decreased compared to baseline and includes four lymphatics that did not display phasic contractions (Fig. 5*H*).

There was also a slight decrease in [Ca^2+^]_i_ just after the Ca^2+^ transients stopped, as the vessel relaxed (Fig. 5*F*). The data summarized from five vessels studied show that this change in “basal” or diastolic [Ca^2+^]_i_ between transient over time was insignificant (Fig. 5*I*). In contrast, normalized EDD increased significantly at 5–7 and 30–32 min after H1152 treatment. Combined, the data indicate that the dilation of mesenteric lymphatic vessels caused by H1152 occurs in the absence of a significant change in diastolic [Ca^2+^]_i_.

**Figure 6:**
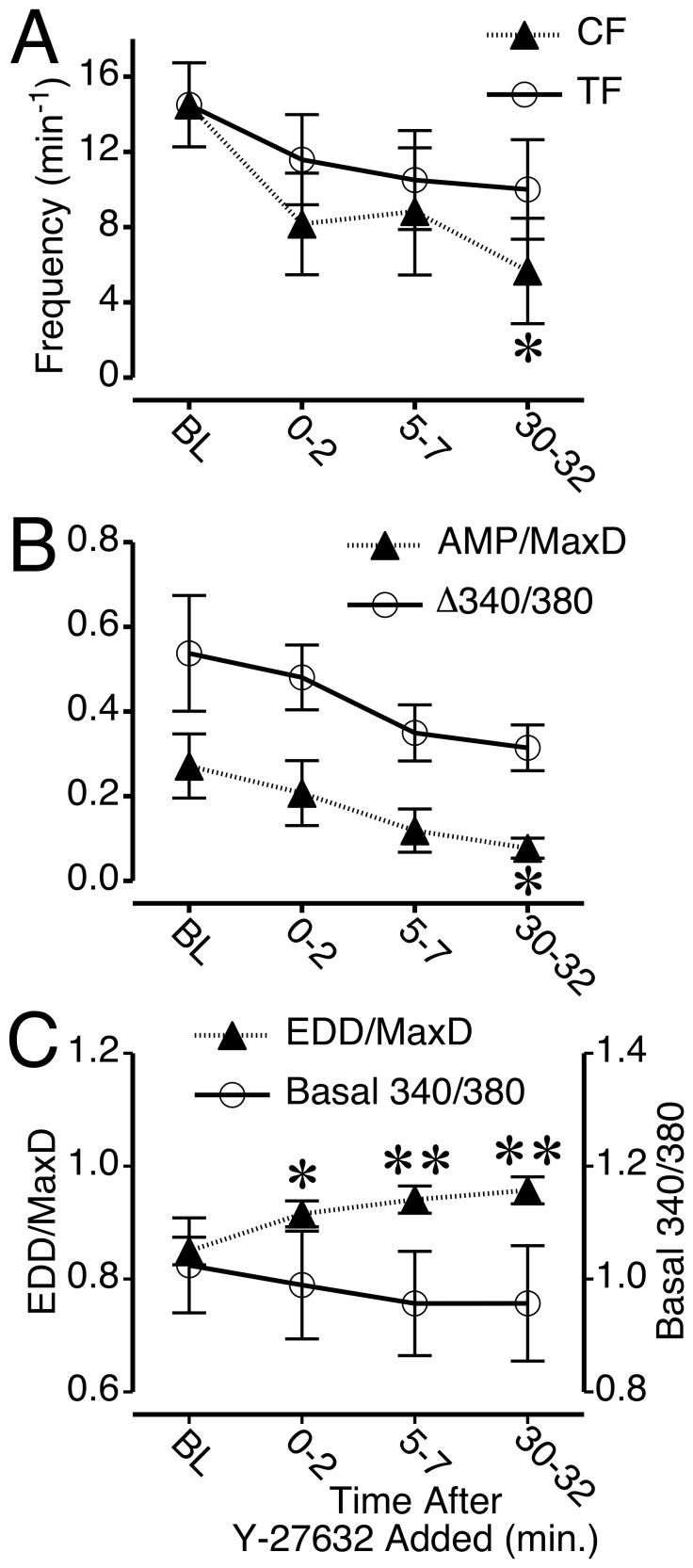
Summary of changes in Ca^2+^ transients, phasic contractions, and tone in rat mesenteric collecting lymphatics treated with Y-27632. *A*. Time course of mean CF and transient frequency (TF) before and during 5 μM Y27632 treatment. *B*. Time course of mean AMP/MaxD and the Ca^2+^ transient magnitude (Δ340/380). *C.* Time course of mean EDD/MaxD and the basal (diastolic) 340/380 ratio between Ca^2+^ before and during Y-27632 treatment. *P<0.05 and **P<0.01 versus baseline. N = 6 lymphatics studied.

Treatment of isolated, fura-2-loaded lymphatics with 50 μM Y-27632 caused similar changes, although with some notable differences. First, while four out of the six vessels tested ceased phasic contractions following the addition of Y-27632, only one ceased during the 0–2 min period after addition, two ceased at 5–7 min, and one stopped at the 30–32 min time point. Thus, the summarized data show a disparity at each time point between CF and TF after Y-27632 was added, however the differences are not significant (Fig. 6*A*). It is worth noting, however, that compared to baseline, the CF was significantly lower than baseline at 30–32 min. after Y-27632 was added, while the TF was not (Fig. 6*A*). Both the mean Ca^2+^ transient magnitude (Δ340/380 ratio) and mean normalized AMP decreased slightly over time, with mean normalized AMP displaying a significant difference from baseline at 30–32 min of Y-27632 treatment (Fig. 6*B*). Like H1152, Y-27632 caused a significant increase in normalized EDD in fura-2-loaded lymphatics (Fig. 6*C*). In contrast, the diastolic, basal [Ca^2+^]_i_ between transients decreased only very slightly, with no significant change from baseline, showing that the upward trend in normalized EDD caused by Y-27632 occurs in the absence of a significant change in the basal, diastolic [Ca^2+^]_i_.

### Constitutively active-ROCK protein transfection increases tone of lymphatics but does not affect Ca^2+^ transients

**Figure 7:**
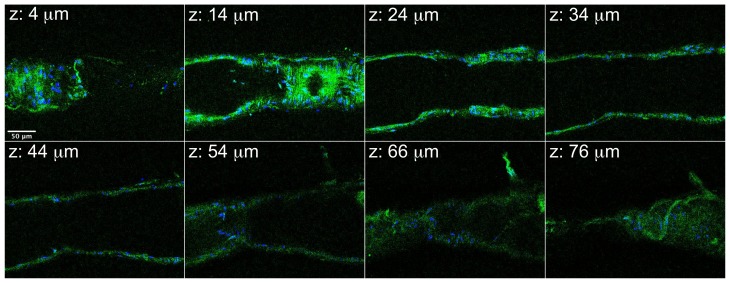
Confirmation of EGFP Protein Transfection in Isolated Lymphatic Vessels. Selected confocal images from a z-stack of 43 images are shown. The distance from the start of the z-stack is shown at the top left of each image. Transfected EGFP protein is shown in green, and nuclei are labeled in blue. Scale bar  =  50 μm. Representative of three separate experiments.

We next tested how experimentally enhancing ROCK in lymphatics may affect pumping, utilizing a protein transfection protocol with isolated lymphatics [18]. The protein transfection mix was added to the abluminal compartment to target the smooth muscle layer. We confirmed the transfection of vessels using a mix of TransIT-LT1 and recombinant EGFP protein, applied for 30 min. (Fig. 7). Controls included applying TransIT-LT1 alone or EGFP alone to lymphatics, and in both cases almost no signal was detected using the excitation/emission channel used for EGFP (data not shown).

**Figure 8:**
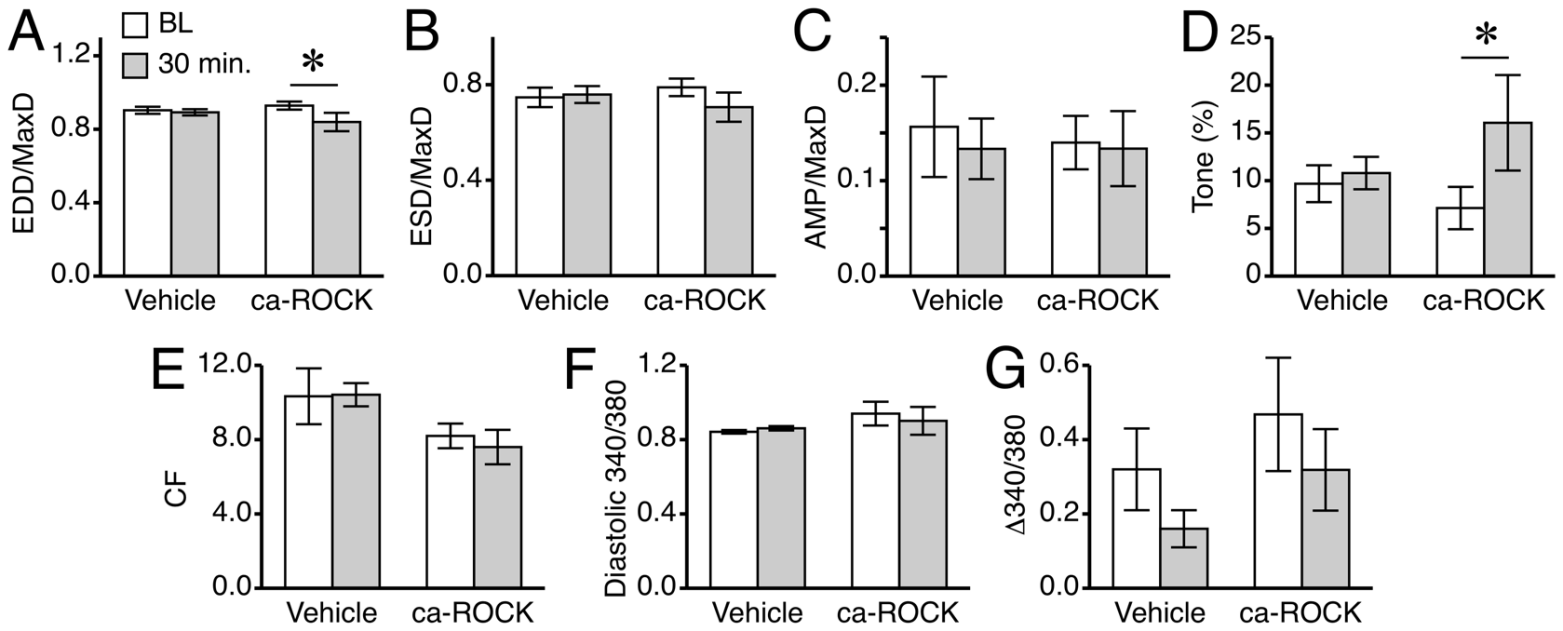
Transfection of lymphatics with ca-ROCK protein increases tone. Isolated, fura-2 loaded rat mesenteric lymphatics were treated with ca-ROCK protein (final concentration 2 μg/ml) combined with TransIT-LT1 (8 μl/ml final), or with the same amount of TransIT-LT1 alone (Vehicle). *A.* Mean EDD/MaxD. *B.* Mean ESD/MaxD. *C.* Mean AMP/MaxD. *D.* Mean tone. *E.* Mean CF. *F.* Mean diastolic 340/380 ratio. *G*. Mean change in the 340/380 ratio during Ca^2+^ transients. *P<0.05 versus baseline (BL), same treatment group. N = 5 vessels for the ca-ROCK group, and N = 6 vessels for the vehicle group.

Transfection of fura-2-loaded isolated lymphatic vessels with ca-ROCK decreased EDD/MaxD and increased its related measure lymphatic tone (Fig. 8*A and D*). However, ca-ROCK did not significantly change basal [Ca^2+^]_i_ between transients (Fig. 8*F*). ESD/MaxD, AMP/MaxD, and CF did not change in response to ca-ROCK treatment (Fig. 8B, C, E). The transfection reagent TransIT appeared to cause a notable decrease, even if statistically insignificant, in the magnitude of Ca^2+^ transients (Δ340/380). Although this raised concern, there was no associated change in AMP/MaxD (Fig. 8*C and G*). Overall, these data indicate that excess ROCK activity leads to an increase in tone in the absence of an increase in [Ca^2+^]_i_.

## Discussion

The current study shows that ROCK1 and ROCK2 are present in lymphatic vessels. In addition, manipulation of ROCK activity can differentially affect [Ca^2+^]_i_ and lymphatic pumping. Pharmacologic inhibition of ROCK predominantly reduces, while experimental enhancement of ROCK increases, lymphatic tone. These changes occurred in the absence of a significant change in the diastolic [Ca^2+^]_i_, suggesting that ROCK promotes lymphatic tone by increasing sensitization of the tonic contractile apparatus to Ca^2+^. To a lesser extent, inhibition of ROCK also negatively impacts phasic contractions in rat mesenteric collecting lymphatic vessels. This appears to be secondary to the decrease in tone, as experimentally increasing ROCK activity does not increase phasic contractions. We also observed that in vessels that ceased phasic contractions following treatment with ROCK inhibitors, Ca^2+^ transients persisted. This finding suggests that a certain minimal ROCK-mediated tone may be needed for the phasic contractions. This was reinforced by the observation that in lymphatics where ROCK inhibitors did not completely cease phasic contractions, the tone was markedly reduced and the phasic contractions often were too weak to elicit a reduction in lymphatic vessel diameter. Taken together, these data demonstrate an important role for ROCK in establishing collecting lymphatic vessel tone, and the importance of ROCK-mediated tone for the phasic contractile cycle in lymphatics.

To our knowledge, this is the first study to show that ROCK1 and ROCK2 are expressed in lymphatic vessels, and to provide detail on localization. ROCK1 was detected mostly in smooth muscle and unidentified cells on the abluminal surface, which could possibly be dendritic cells, vasa vasorum, or mast cells [23]–[26]. In contrast, ROCK2 was detected in smooth muscle and endothelium. ROCK has been widely studied in vascular and other smooth muscle types, and the two isoforms of ROCK participate in a variety of smooth muscle cell functions relating to cell contraction, shape, and motility. While some distinct molecular interactions for the two ROCK isoforms have been described, both ROCK1 and ROCK2 knockout mice survive and are fertile, suggesting enough redundancy to compensate for loss of one or the other isoform [27],[28]. Our new evidence that ROCK1 and ROCK2 are expressed in lymphatic smooth muscle support the validity our pharmacologic data.

A few previous studies have also implicated a role for ROCK in lymphatic pump function. Hosaka et al demonstrated that 1–6 μM Y-27632 could increase EDD and stop pumping of isolated rat iliac collecting lymphatics [16]. In their study, they also observed that Y-27632 could elicit relaxation when the lymphatic vessels were preconstricted with norepinephrine, the prostaglandin H2 analog U-46619, or a high KCl bath solution. They also showed that removal of the endothelial layer did not inhibit the Y-27632-induced relaxation of lymphatic vessels [16]. In a more recent study, Si et al reported that Y-27632 reduced tone, contraction frequency, contraction amplitude, and fractional pump flow of isolated rat thoracic ducts [17]. In their study, they also studied the impact of hemorrhagic shock on thoracic duct pumping and RhoA protein and phosphorylation levels. They found that in thoracic ducts isolated from rats that had undergone a fixed pressure hemorrhage for 30 min., RhoA protein levels were elevated and there was increased phosphorylation of RhoA. This was followed by a decline in RhoA levels after 2 h of hemorrhagic shock, that was accompanied by decreased CF and tone [17]. In our own study of how alcohol intoxication depresses the myogenic responsiveness of lymphatic vessels, we observed a decrease in the active, GTP-bound form of RhoA in lymphatic vessels isolated from the alcohol-intoxicated rats, compared to controls [18]. We also reported that transfection of lymphatics from the alcohol-intoxicated rats could restore the myogenic response to step elevations in luminal pressure [18]. Our data in the current study support the findings of these previous reports. In addition, we extend these findings by showing that a structurally distinct ROCK inhibitor produces similar results. In addition, a higher concentration of Y-27632 was required to cease phasic contractions in isolated mesenteric lymphatics than that reported for the rat iliac lymphatics [16]. Also, in the current study not all lymphatics stopped pumping even with the highest concentration of Y-27632 used, suggesting differences based on which tissue bed from which the collecting lymphatics are isolated [29]. Differences in the rat strain or in the experimental protocols may also be accountable, as in the current study we used a lower luminal pressure and no imposed luminal flow. With the assumption that the pharmacological inhibitors utilized have reasonable specificity toward ROCK, our data and the previous reports support that endogenous ROCK has a role in the establishment of tone in lymphatic vessels.

Unlike the previous studies investigating the role of ROCK using inhibitors, we also directly tested whether we could produce a gain of ROCK-mediated function, by transfecting isolated lymphatic vessels with purified ca-ROCK protein. In previous studies, we investigated the inhibition of upward luminal pressure step-induced myogenic constriction in lymphatic vessels isolated from rats that had undergone an acute alcohol intoxication protocol [18],[21]. Application of ca-ROCK to those lymphatic vessels helped restore their myogenic responsiveness [18]. However, in that study we did not directly investigate how ca-ROCK can modulate tone in the absence of the pressure step protocol. Another notable difference between the current study and the previous alcohol intoxication study is that all of the rats used in the previous study had undergone surgery to implant gastric catheters 2–3 days prior to the isolation of the lymphatics. In the current study, we observed that transfection of ca-ROCK increases tone, providing additional evidence that ROCK plays an important role in the development of tone in rat mesenteric collecting lymphatics. On the other hand, transfection with ca-ROCK did not affect phasic contractions or AMP. Alone, this negative result is not conclusive about ROCK's role in phasic contractions. However, considering that inhibition of ROCK decreased both tone and CF, while increasing ROCK activity increased only tone, an attractive scenario is that a certain basal tone, requiring normal ROCK activity, is necessary for phasic contractions to occur.

Additionally, novel insights were gained with our studies of how inhibition or enhancement of ROCK affects [Ca^2+^]_i_ in lymphatics. An interesting set of findings from our study is that in several lymphatics studied, inhibition of ROCK, particularly with H1152, prevented lymphatic phasic contractions while Ca^2+^ transients persisted. The total loss of phasic contractions in the first two minutes after H1152 addition, while Ca^2+^ transients retained a magnitude that was comparable to baseline, suggests a role for ROCK in the mechanism by which the lymphatic phasic contractile apparatus senses changes in [Ca^2+^]_i_. Over time, the lymphatics also became more relaxed, with an increased EDD. There was also a very slight decrease in diastolic [Ca^2+^]_i_, however this insignificant change appears to be too small to account for the significant relaxation. In the case of Y-27632 treatment, the cessation of phasic contractions occurred more variably, but the increase in EDD in the absence of a significant change in diastolic [Ca^2+^]_i_ was consistently observed. This differences between the H1152 and Y-27632 are probably based on distinct structures and binding to the ROCK1 and ROCK2 catalytic domains. Transfection with ca-ROCK increased lymphatic tone in the absence of a change in diastolic [Ca^2+^]_i_. For comparison, we previously observed that step increases in luminal pressure caused small, yet significant elevations in diastolic [Ca^2+^]_i_ associated with increased lymphatic tone [6],[18]. In addition, we observed that intoxication of rats with alcohol impaired the ability of their mesenteric lymphatics to constrict in response to upward pressure steps, yet the increases in calcium were not only still present, but were amplified. Transfection with ca-ROCK rescued the myogenic constriction response [18],[21]. In the current study, transfection of ca-ROCK did not produce an increase in CF or AMP. This data suggests that ROCK primarily affects tone. One potential problem we noticed was that the transfection protocol (with or without ca-ROCK) produced a noticeable decrease over time in the Ca^2+^ transient magnitude. However, AMP was not decreased over the same time period. This may indicate that prior to the transfection, the amount of Ca^2+^ released from the SR just prior to each contraction is in excess of what is required to elicit a contraction in smooth muscle, but might also indicate interference with the detection of the transients. Additional work will be needed to determine the cause.

Very recent evidence has also emerged indicating that activation of PKC also can increase the Ca^2+^ sensitivity of the lymphatic contractile mechanism [30]. The study suggested that both MLC-dependent and MLC-independent mechanisms contribute to the Ca^2+^ sensitivity, as initial rise in tension of isolated permeabilized lymphatics elicited by phorbol esters was accompanied by elevated phosphorylation of MLC, but the later, steady-state increase in Ca^2+^ sensitivity were not [30]. Whether or not these PKC activation-mediated Ca^2+^-sensitizing mechanisms involve ROCK, or represent a separate, parallel pathway, remains to be determined.

It is important to note that we did not remove the lymphatic endothelium in all of our studies, making it possible that inhibition or enhancement of ROCK activity in the endothelium may have contributed to the overall observed results. However, we feel this is unlikely given the previous report that Y-27632 caused lymphatic relaxation when the endothelium was removed [16]. In addition, when we transfected lymphatic vessels with EGFP protein, we observed uptake of EGFP in the outer, smooth muscle layer. Based on this result, we feel it is a fairly safe assumption that the transfected ca-ROCK protein was also predominantly present in the smooth muscle layer.

We also loaded the lymphatics with fura-2 from the abluminal compartment (no fura-2 AM was added to the lumen) to target its loading to the smooth muscle layer. The luminal compartment had a pressure of 2 cm H_2_O while the height of the bath was less than 0.5 cm H_2_O, a sufficient hydrostatic gradient to restrict movement of dye toward the lumen. However, with lymphatics having a relatively thin smooth muscle layer, it is possible that the 340/380 ratios we observed include [Ca^2+^]_i_ detected in endothelial cells as well. Dendritic cells, vasa vasorum, and other yet-to-be identified cell types have also been observed in the collecting lymphatic architecture [23],[24]. Ca^2+^ detected within these cells could also potentially have contributed to the overall 340/380 ratios we detected, despite our best efforts to avoid such areas. Also, we did observe a slight increase in EDD caused by fura-2-loading of the lymphatic vessels. A possible explanation is buffering of Ca^2+^ from fura-2, which was reported to reduce peak force of muscle twitches in skeletal and cardiac muscle [31],[32].

Pharmacologic inhibition of ROCK has been utilized as a clinical strategy to prevent cerebral vasospasm for nearly two decades [33]. Additional investigations implicating the RhoA/ROCK pathway in the pathogenesis of hypertension, coronary vasospasm, stroke, atherosclerosis, heart failure, and diabetes indicate its potential role as an important therapeutic target. Several pharmaceutical companies are already actively engaged in the development of ROCK inhibitors as the next generation of therapeutic agents for these diseases [34]. Our study indicates that therapeutic strategies to inhibit ROCK should consider the potential impact on lymphatic pump function.

In summary, our data suggests a role for ROCK in the Ca^2+^-sensitizing mechanism for the development of tone in mesenteric collecting lymphatics. In addition, ROCK inhibitors can also have a profound effect on the phasic contractions responsible for the pump function of collecting lymphatics. These data also highlight important, unanswered questions, such as what myosin isoforms are present in lymphatics and what are their sensitivities toward [Ca^2+^]_i_ or ROCK; which of these myosin isoforms are involved in tonic versus phasic contractions; and what degree of cooperation may there be between myosin isoforms in both tonic and phasic contraction generation.

## Supporting Information

Figure S1 **Representative tracing of an untreated isolated rat mesenteric lymphatic.** The vessel was kept at 2 cm H_2_O during the entire protocol, and is representative of three separate experiments.
(TIF)

Figure S2 **Concentration-response of lymphatic contractile parameters in response to H1152.** All data are normalized to the maximum responses observed.
(TIFF)

Figure S3 **Concentration-response of lymphatic contractile parameters in response to Y-27632.** All data are normalized to the maximum responses observed.
(TIFF)

Movie S1 **Confocal image stack of an isolated rat mesenteric collecting lymphatic labeled for ROCK1.** Confocal slices were obtained in single photon mode at 2 μm intervals, which are indicated at the top left of each frame. Images of individual channels for ROCK1 (green) and nuclei, (white), and an overlay of these two channels are shown in the top row. Images of the glycocalyx (red) and smooth muscle (SM) actin (blue) channels, and an overlay are shown in the left column. All other combinations of overlays fill out the remaining rows and columns. In the bottom red/white overlay (bottom left), magenta areas indicate overlap. Just to the right, in the red/blue/white overlay, the endothelium and smooth muscle layers can be distinguished further due to the orientation of the nuclei. Endothelial nuclei are located in the inner layer and are oriented longitudinally. Smooth muscle nuclei are elongated and oriented perpendicular to the vessel, in the same fashion as the smooth muscle cells. In the green/red images, yellow pixels indicate overlap. In the green/blue images, cyan pixels indicate overlap. In the green/red/blue image, grey/white pixels indicate overlap. ROCK1 labeling overlaps with some areas of smooth muscle, to a lesser extent in the endothelial layer, and is also present within vasa vasorum on the outer surface of the lymphatic vessel. The labeling becomes weaker at the far end of the z-stack because no correction for z-distance was used in the image capturing. This vessel is representative of three separate experiments.
(AVI)

Movie S2 **Confocal image stack of an isolated rat mesenteric collecting lymphatic labeled for ROCK2.** Confocal slices were obtained in single photon mode at 2 μm intervals, which are indicated at the top left of each frame. Images of individual channels for ROCK2 (green) and nuclei, (white), and an overlay of these two channels are shown in the top row. Images of the glycocalyx (red) and smooth muscle (SM) actin (blue) channels, and an overlay of these two channels are shown in the left column. All other combinations of overlays fill out the remaining rows and columns. In the red/blue overlay, magenta pixels indicate overlap. In the green/red overlay, yellow pixels indicate overlap. In the green/blue overlay, cyan pixels indicate overlap. In the green/red/blue image, grey/white pixels indicate overlap. Overlays including nuclei are also included to help distinguish the endothelial and smooth muscle layers. Strong ROCK2 labeling is evident in the smooth muscle layer and endothelium, and is also present within the vasa vasorum on the outer surface of the lymphatic vessel. The labeling becomes weaker at the far end of the z-stack because no correction for z-distance was used in the image capturing. This vessel is representative of three separate experiments.
(AVI)

Movie S3 **Time-lapse 340/380 ratio images of a pumping mesenteric collecting lymphatic during baseline.**
(AVI)

Movie S4 **Time-lapse 340/380 ratio images of a pumping mesenteric collecting lymphatic immediately after adding 10 μM H1152.**
(AVI)
