# Supplementary figures and images for: Rho Kinase Enhances Contractions of Rat Mesenteric Collecting Lymphatics
Source: PLoS One. 2014 Apr 7;9(4):e94082. doi: 10.1371/journal.pone.0094082 (PMC3978029; doi:10.1371/journal.pone.0094082)

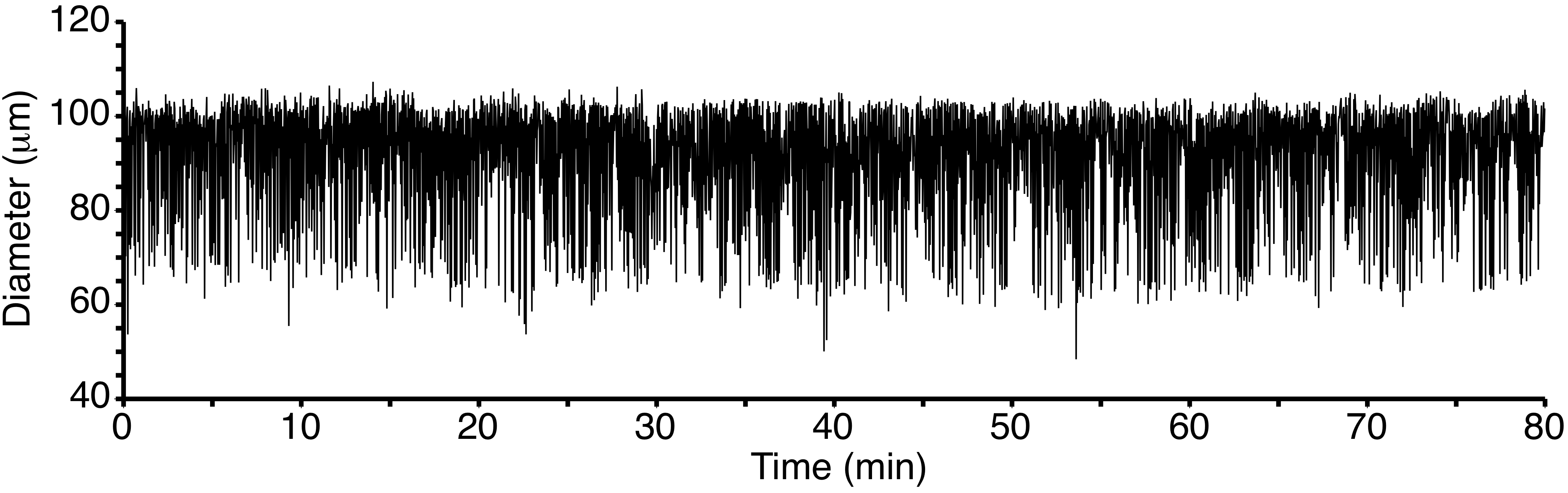

Supplement: Figure S1 — Representative tracing of an untreated isolated rat mesenteric lymphatic. The vessel was kept at 2 cm H2O during the entire protocol, and is representative of three separate experiments. (TIF) [file pone.0094082.s001.tif]

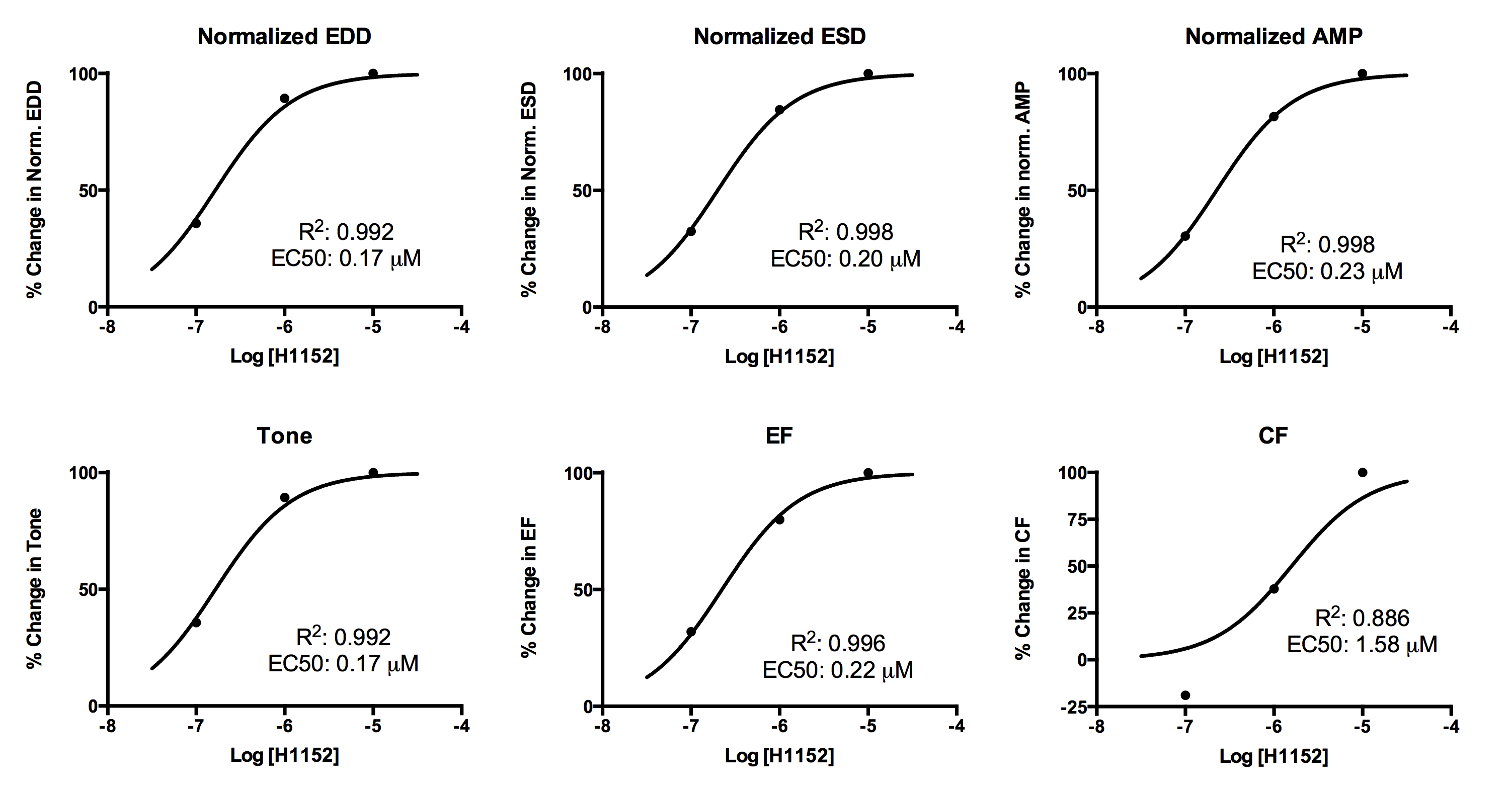

Supplement: Figure S2 — Concentration-response of lymphatic contractile parameters in response to H1152. All data are normalized to the maximum responses observed. (TIFF) [file pone.0094082.s002.tiff]

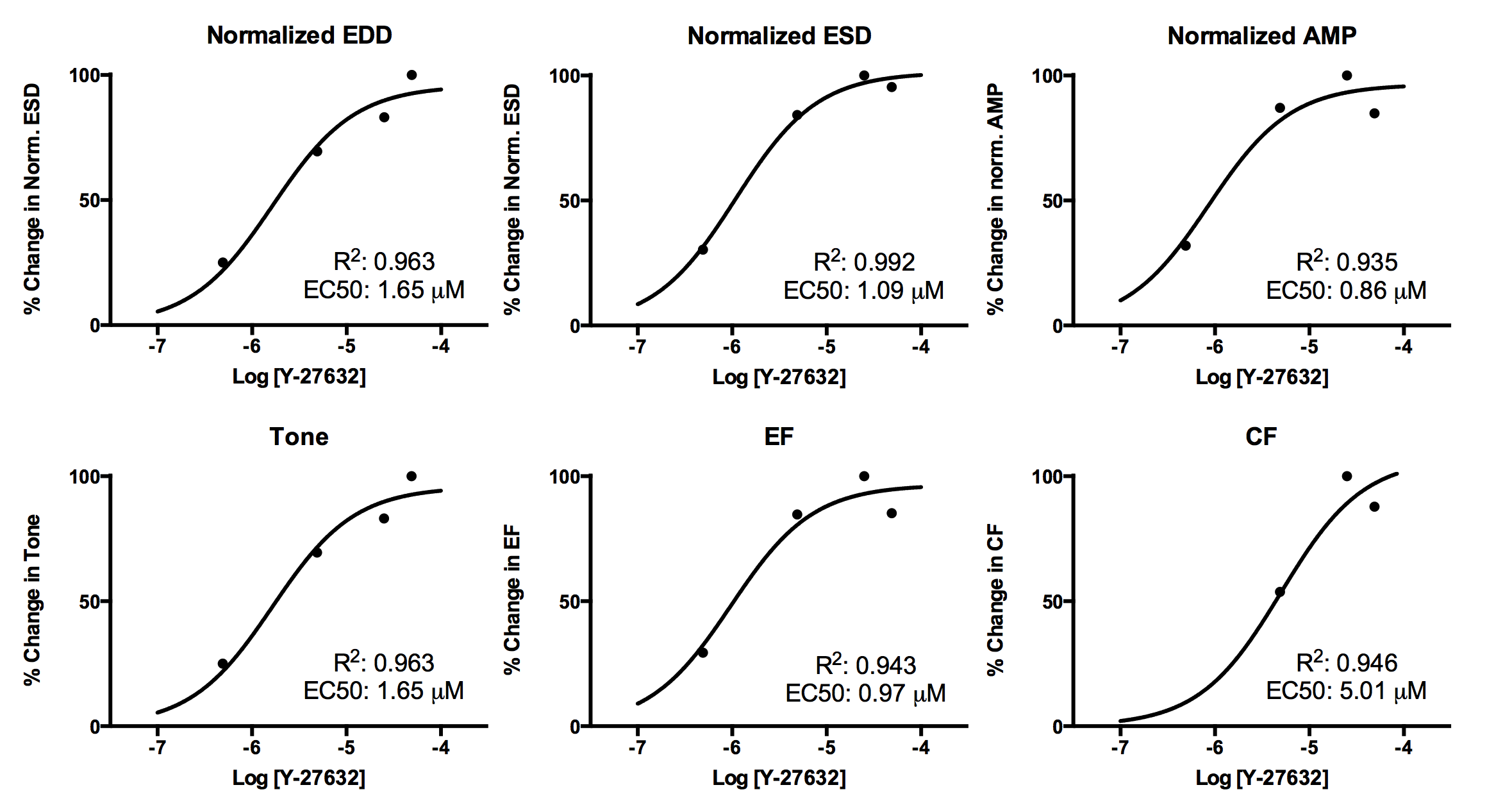

Supplement: Figure S3 — Concentration-response of lymphatic contractile parameters in response to Y-27632. All data are normalized to the maximum responses observed. (TIFF) [file pone.0094082.s003.tiff]
